# Red blood cell agglutination caused by ceftriaxone and its effect on erythrocyte parameters: a case report

**DOI:** 10.11613/BM.2025.011002

**Published:** 2024-12-15

**Authors:** Petra Andrasic, Renata Zrinski Topic, Ivan Pavic, Jasna Lenicek Krleza

**Affiliations:** 1 Department of Medical Biochemistry and Hematology, Children’s Hospital Zagreb, Zagreb, Croatia; 2 Department of Pulmonology, Allergology, Immunology and Rheumatology, Children’s Hospital Zagreb, Zagreb, Croatia; 3 School of Medicine, University of Split, Split, Croatia; 4 University Department of Nursing, Catholic University of Croatia, Zagreb, Croatia

**Keywords:** agglutination, ceftriaxone, hemolytic anemia, preanalytical errors, red blood cell count

## Abstract

Ceftriaxone, a widely used antibiotic, is one of the most common drugs to cause drug-induced immune hemolytic anemia. In this report, we describe the effect of ceftriaxone on red blood cell parameters (low red blood cell count, low hematocrit, and high erythrocyte index values) in two pediatric patients without clinical symptoms of hemolytic anemia. Although automated hematology analyzers have helped to detect incorrect results, a peripheral blood smear examination was necessary for recognizing the erythrocyte agglutinins caused by ceftriaxone. Serological testing was not possible, but the resulting drug-induced antibodies mimicked cold agglutinins in the first patient and warm agglutinins in the second patient. Timely reactions and corresponding laboratory procedures prevented potential complications due to drug administration. This report aims to present laboratory findings and preanalytical challenges in these cases and share our experiences in solving them.

## Graphical abstract

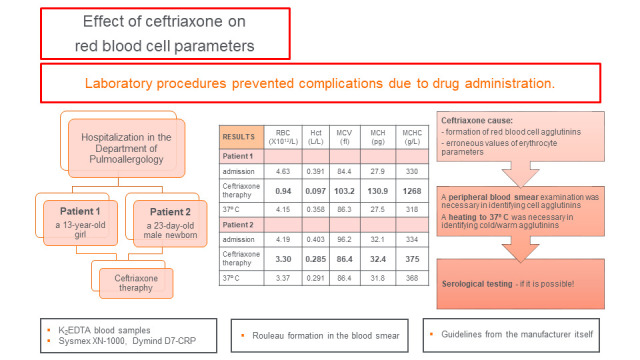

## Highlights

• Ceftriaxone led to the formation of red blood cell agglutinins

• Ceftriaxone causes erroneous values of red blood cell parameters

• A peripheral blood smear was necessary to identify cell agglutinins

• Laboratory procedures prevented complications due to drug administration

• Cessation of ceftriaxone is crucial in preventing life-threatening complications

## Introduction

Ceftriaxone is a third-generation cephalosporin used in the treatment of various bacterial infections like pneumonia, meningitis, and urinary tract infections. This broad-spectrum antibiotic is widely used in pediatric patients of all ages, while also being one of the most common drugs to cause drug-induced immune hemolytic anemia (DIIHA) (*1*, *2*).

Drug-induced immune hemolytic anemia is one type of autoimmune hemolytic anemia (AIHA) wherein extra- or intravascular hemolysis ensues because of a drug-induced immune reaction (*1*, *3*). The most prominent groups of drugs implicated in DIIHA are antibiotics such as penicillins or cephalosporins, anti-inflammatory drugs, and antineoplastic drugs. According to the current research and recorded cases, azithromycin does not cause DIIHA (*3*, *4*). The clinical manifestations are diverse, ranging from mild to severe, depending on the degree of hemolysis as well as antibody class and characteristics (antibody concentration, antigen affinity, thermal amplitude, *etc.*). Ceftriaxone-induced immune hemolytic anemia (CIIHA) is a rare nevertheless potentially life-threatening complication of ceftriaxone therapy, so an early and precise diagnosis is crucial (*2*). It manifests in children who commonly have underlying chronic hematologic or immunological disorders and recurrent or chronic infections (*5*). The prognosis and clinical picture are usually worse in children than in adults. In adults, CIIHA is normally seen within days to weeks of exposure to new drugs, while hemolysis in pediatric patients occurs in a range from 5 to 120 minutes of ceftriaxone administration (*1*-*3*).

Although the pathogenesis of DIIHA has not been completely resolved, several theories causing DIIHA have been presented. It is understood that a complex mechanism of molecular mimicry is involved in DIIHA, like in infections, which implies a cross-reactivity between red blood cell (RBC) antigens and exogenous antigens (*6*). Antibodies in CIIHA are primarily IgM class, responsible for complement activation and intravascular hemolysis, accompanied by the IgG class.

By the mechanism of molecular mimicry, DIIHA may mimic warm or cold antibody AIHA, hereditary hemolytic anemias (*e.g*., hereditary spherocytosis), and other drug-induced hemolysis like in glucose-6-phosphate dehydrogenase deficiency (*7*). Warm agglutinins are autoantibodies typically activated at body temperatures of 37 °C or higher, binding to RBC and destroying them prematurely. Most warm agglutinin immune hemolysis is extravascular without complement activation (*4*, *8*). Cold agglutinins are monoclonal or polyclonal antibodies activated at low temperatures and lead to autoagglutination of the RBC membrane (*8*, *9*). Cooling of acral parts of the body allows the cold agglutinins to bind to the erythrocytes and induce the complement *via* the classical pathway. Antibodies detach from the antigen-antibody complex when the blood temperature rises to approximately 37 °C (*10*).

Two cases of RBC agglutination caused by ceftriaxone in patients of pediatric age and corresponding laboratory procedures are presented below. This report aims to present the laboratory findings and preanalytical challenges in these cases and share our experiences in solving them.

## Case report

**Patient 1:** a 13-year-old girl with no significant medical history, apart from a tonsillectomy at the age of three, was evaluated in the emergency room based on the symptoms of syncope, fever, and cough. It was decided that the patient should be admitted to the Department of Pulmoallergology, for right-sided pneumonia. The observed RBC results are shown in Table 1 (admission). There were no abnormal or suspicious findings, nor did the analyzer display any flags. Parenteral antibiotic therapy with ceftriaxone and oral antibiotic therapy with azithromycin were administered to the patient, along with parenteral rehydration and other supportive and symptomatic measures. From the start of hospitalization, the patient’s general condition, together with the auscultation of the lung, gradually improved. Three days after admission, the new blood samples were delivered to the laboratory for control monitoring of patient values. Red blood cell count and hematocrit (Hct) were unexpectedly low and disproportionate to hemoglobin (Hb) concentration.

**Table 1:** The results of red blood cell parameters and presented messages by analyzers in different samples from Patient 1 during the hospitalization

**Parameter**	**Day 1 Admission**	**Day 4 Sample 1.0** ^α^	**Day 4 Sample 1.1** ^β^	**Day 4 Sample 1.2^γ^**	**Day 4 Sample 1.3** ^δ^	**Day 4 Sample 2.0 ^ε^**	**Day 4 Sample 2.1^ζ^**
RBC (x10^12^/L)	4.63	0.94*	1.52*	2.44?	3.77	3.23*	4.15
Hb (g/L)	129	123*	125*	126?	124*	115*	114
Hct (L/L)	0.391	0.097*	0.154*	0.239?	0.339	0.304*	0.358
MCV (fL)	84.4	103.2*	101.3*	97.9?	89.9	94.1*	86.3
MCH (pg)	27.9	130.9*	82.2*	51.4?	32.9*	35.6*	27.5
MCHC (g/L)	330	1268*	812*	525?	366*	378*	318
RDW-CV (%)	12.6	----	----	14.0?	15.9	16.4*	12.9
**Message (analyzer)**							
RBC Abn Distribution (Sysmex XN-1000)	-	+	+	/	-	-	-
Dismorphic Population (Sysmex XN-1000)	-	+	+	/	-	-	-
RBC Agglutination (Sysmex XN-1000)	-	+	+	/	-	-	-
Turbidity/HGB Interf? (Sysmex XN-1000)	-	+	+	/	+	+	-
HGB Abnor./Interfere? (Dymind D7-CRP)	/	/	/	+	/	/	/
^α^First sample three days after admission measured by Sysmex XN-1000 (impedance). ^β^First sample measured by Sysmex XN-1000 (fluorescence flow cytometry). ^γ^First sample measured by Dymind D7-CRP (impedance). ^δ^First sample after heating at 37 °C for 60 minutes measured by Sysmex XN-1000 (fluorescence flow cytometry). ^ε^Second sample (repeated collection after three hours) measured by Sysmex XN-1000 (fluorescence flow cytometry). ^ζ^Second sample incubated at 37 °C for 60 minutes measured by Sysmex XN-1000 (fluorescence flow cytometry). *Asterisks from analyzer. Symbol ‘+’ means the message was displayed on the analyzer. Symbol ‘-’ means the message was not displayed on the analyzer. Symbol ‘/’ means the sample was not measured on the analyzer that throws out this message. Symbol ‘?’ next to the analysis result means the result is suspicious by the Dymind analyzer. RBC - red blood cells. Hb - hemoglobin. Hct - hematocrit. MCV - mean corpuscular volume. MCH - mean corpuscular hemoglobin content. MCHC - mean corpuscular hemoglobin concentration. RDW-CV - red cell distribution width-coefficient of variation.							

**Patient 2:** a 23-day-old male newborn with a blocked nose and fever was hospitalized for acute bronchiolitis in the Department of Pulmoallergology one month later after receiving Patient 1. Patient 2 tested positive for respiratory syncytial virus (RSV) and SARS coronavirus 2 withal. During the third and fourth days of hospitalization, new control samples were sent to the laboratory for patient monitoring and analyzed on Sysmex XN-1000 (Table 2, 3rd day and 4th day). Parenteral antibiotic therapy with ceftriaxone was initiated on the fourth day of hospitalization. The patient had been afebrile since the third day after ceftriaxone admission, while the day later a new blood sample was again sent to the laboratory for hematological processing. The results then showed lower hemoglobin (Hb) concentration and hematocrit (Hct) and higher mean corpuscular hemoglobin concentration (MCHC). After a change in therapy and a clean auscultatory finding of the lungs, the patient was discharged from the hospital, and laboratory monitoring was continued in the competent institution.

**Table 2:** The results of red blood cell parameters and presented message by analyzer in different samples from Patient 2 during the hospitalization

**Parameter**	**Admission^†^**	**3rd day**	**4th day^Δ^**	**7th day**	**7th day^‡^**
RBC (x10^12^/L)	4.19	3.80	3.93	3.30	3.37
Hb (g/L)	135	125	122	107*	107*
Hct (L/L)	0.403	0.352	0.345	0.285	0.291
MCV (fL)	96.2	92.6	87.8	86.4	86.4
MCH (pg)	32.1	32.9	31.0	32.4*	31.8*
MCHC (g/L)	334	355	354	375*	368*
RDW-CV (%)	16.2	13.9	13.6	13.7	13.7
**Message on analyzer**					
Turbidity/ HGB Interf?	/	-	-	+	+
^†^The only sample measured by Dymind D7-CRP. ^Δ^Ceftriaxone administration was started. ^‡^Sample after incubation at 37 °C for 60 minutes. *Asterisks from analyzer. Symbol “+” means the message was displayed on the analyzer. Symbol “-” means the message was not displayed on the analyzer. Symbol “/” means the sample was not measured on the analyzer that throws out this message. RBC - red blood cells. Hb - hemoglobin. Hct - hematocrit. MCV - mean corpuscular volume. MCH - mean corpuscular hemoglobin content. MCHC - mean corpuscular hemoglobin concentration. RDW-CV - red cell distribution width-coefficient of variation.					

Informed consents were obtained from the legal guardians of each patient.

## Laboratory analyses

Peripheral blood samples taken for a complete blood count (CBC), obtained by a butterfly phlebotomy technique in a 2.0 mL Vacuette tube (K_2_EDTA, Greiner Bio-One, Kremsmünster, Austria), were analyzed on Sysmex XN-1000 (Sysmex Corporation, Kobe, Japan) and Dymind D7-CRP (Shenzhen Dymind Biotechnology Co., Shenzhen, China) hematology analyzers with supporting reagents. A capillary blood sample, collected by pricking the skin with a 1.5 mm width and 2.0 mm depth lancet (Greiner Bio-One, Kremsmünster, Austria) in a 1.0 mL test tube (K_2_EDTA, Kabe Labortechnik, Nümbrecht-Elsenroth, Germany), was analyzed on Dymind D7-CRP.

Routine laboratory tests were requested as part of diagnostic processing for Patient 1. The results of hematology parameters are presented in Table 1, Sample 1.0. Red blood cell index values, including mean corpuscular volume (MCV), mean corpuscular hemoglobin content (MCH) and MCHC, were extremely high, with one exception: red cell distribution width-coefficient of variation (RDW-CV) could not be measured. Previously mentioned RBC parameters were flagged, which indicates inaccurate results. The analyzer gave the messages as follows: ‘RBC Abn Distribution‘, ‘Dismorphic Population‘, ‘RBC Agglutination‘, and ‘Turbidity/HGB Interf? ‘, pointing to erythrocyte agglutination. Additionally, the sample tube had no evidence of hemolysis, icterus, or lipemia, nor were any aggregates or clumps visually observed. Complete blood count testing was repeated. However, the measurement principle on the analyzer was substituted, from direct current detection method (impedance) to fluorescence flow cytometry. The RBC results did not vary greatly (Table 1, Sample 1.1), and the same messages from the analyzer were generated. Next, the sample was analyzed on another analyzer, Dymind D7-CRP. The values of RBC parameters were displayed with question marks and the analyzer’s message: ‘HGB Abnor./Interfere?‘ indicating unreliable results (Table 1, Sample 1.2).

The RBC results of Patient 2 on admission were in the reference interval according to age (Table 2, admission). The following RBC parameters Hb, MCH, and MCHC were flagged, and the analyzer displayed the message ‘Turbidity/HGBInterf?‘ (Table 2, 7th day). The possibility of hemolysis, icterus, or lipemia interference was excluded along with the presence of aggregates by visual examination and automated detection.

## Interventions

**Figure 1:**
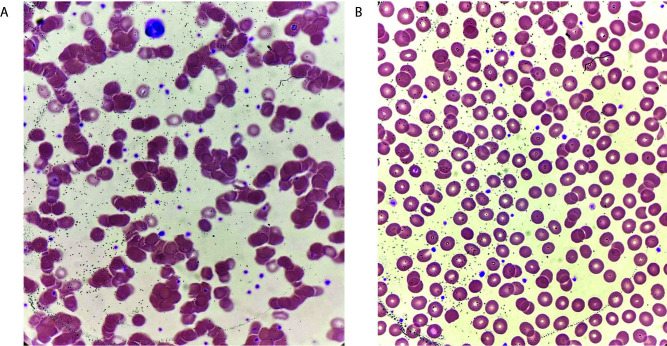
Peripheral blood smear, RAL Stainer MCDh, 1000x. A: Sample from Patient 1 collected three days after admission presenting with erythrocyte clusters in the Rouleau formation. B: Sample from Patient 1 after repeated collection and incubation at 37 °C for 60 minutes without showing erythrocyte clusters. Photos are originally by the author.

In the following step of the first case, a peripheral blood smear was prepared using an automated staining device RAL Stainer MCDh (RAL Diagnostics, Martillac, France) with supporting reagents. When the blood smear was examined under the optical microscope (Olympus BX41, Olympus, Tokyo, Japan), clusters of RBCs were revealed in each field, in the Rouleau formation (Figure 1A). Due to suspicion of cold agglutinins, the tube was heated at 37 °C for 60 minutes, according to guidelines from the manufacturer, in a digital water bath, SWB6D with BioCote antimicrobial protection (Stuart Equipment, Staffordshire, England). The results received after the heating (Table 1, Sample 1.3) seemed more logical, and the RDW-CV could be measured. However, the RBC parameters were still marked with asterisks and the analyzer’s flag: ‘Turbidity/HGB Interf?‘. The clinician was contacted, and through conversation, detailed the patient’s current therapy. In addition, there were no clinical manifestations of bleeding in the patient. It was agreed upon sending a new sample to the hematology laboratory and to stop using ceftriaxone in therapy. Between the first sampling at 8 a.m. and the second sampling the same day at 11 a.m., the patient received a dose of ceftriaxone at 9 a.m., just before a call from the laboratory, after which an infusion was administered up until 10:30 a.m. The received sample was run on Sysmex XN-1000 hematology analyzer and the results were, again, similar to the previous ones, with asterisks and a message from the analyzer (Table 1, Sample 2.0). Following the latest results, the test tube was incubated at 37 °C for 60 minutes. The RBC values, after incubation for 60 minutes, seemed rectified, without any asterisks or flags (Table 1, Sample 2.1). Moreover, the peripheral blood smear did not contain erythrocyte clusters (Figure 1B).

In the second case, the clinician confirmed ceftriaxone therapy, and drug effect on hematological parameters were spotted again. Therefore, the use of ceftriaxone was stopped, and the therapy was changed after the clinician was contacted. Fortunately, at that time there were still no clinical signs indicating bleeding in the patient. The sample tube was incubated at 37 ºC for 60 minutes, and the results were almost identical to those before heating, pointing to warm agglutinins (Table 2, 7th day).

## What happened?

Using ceftriaxone in therapy led to the formation of RBC agglutinins that behaved as cold agglutinins in the first case and as warm in the second. Consequently, the RBC parameters were erroneous. The incorrectly measured RBC count and Hct were reflected in the calculated RBC indices. Both patients were without clinical symptoms of bleeding, but Patient 2 had a lower hemoglobin concentration after drug administration.

## Discussion

We presented here two cases of drug-induced immune hemolytic anemia, with the onset of cold and warm agglutinins, respectively. Serological testing was not possible, but the resulting drug-induced antibodies mimicked cold agglutinins in the first patient and warm agglutinins in the second. Coombs’ direct antiglobulin test (DAT) in CIIHA is mostly positive for the C3 component of complement and, in some cases, for IgG, as was shown in the study by Arndt *et al.*, where 100% of samples were reactive with anti-C3 and 47% were reactive with anti-IgG, though negative DAT has been reported too (*1*, *11*). Mechanisms of DIIHA that can present as warm autoantibodies are cross-reactive autoantibody production and immunoglobulin adsorption, which were described in the article by Loriamini *et al.* (*12*). Besides these two, ceftriaxone also acts by neoantigen-dependent and hapten-specific mechanisms (*4*, *12*).

The above-mentioned agglutinins may cause false laboratory findings. Created microaggregates could be counted as leukocytes or single erythrocytes by hematology analyzers, causing false CBC results, with the bigger ones not even being possible to be classified. The RBC counts are decreased, and MCV and RDW-CV are falsely elevated or not measurable from the RBC histogram. Errors are also observed in Hct, MCH, and MCHC since these parameters are ultimately calculated using RBC counts or MCV (*9*, *13*). Such deviations in values were noticed in the first presented case. Due to RBC agglutination, both MCH and MCHC were much higher while Hct was spuriously low. Higher MCHC and lower Hct, only less dramatic, were recognized in the second case too. Hematocrit can also be measured directly from the cumulative value of the individual cell pulse heights, as it is on Sysmex analyzers. However, even such a method is not resistant to the presence of agglutinins (*14*, *15*). Effects of cold agglutinins may be visible on platelets (Plt), so incorrect Plt counts and mean platelet volume (MPV) are not unusual (*9*). Similar errors in RBC values due to cold agglutinins were reported by Topic *et al.* (*16*). Since it is known cold agglutinins separate from the complex at 37 °C, it is possible to overcome these analytical problems by warming the sample to 37 °C before analysis (*9*, *10*). In a case report submitted by Yasar *et al.*, a female patient with cold agglutinin disease had incorrect CBC results that were improved after heating the sample to 37 °C (*9*). An interesting point for future investigations can be observed in a published report in which Daves *et al.* concluded that in a certain number of samples, but not in all, the reticulocyte channel heated to 41 °C can quickly and effectively correct CBCs without preheating the sample in comparison with the impedance method (*17*). In our cases, the fluorescence flow cytometry did not correct CBC parameters. Flags from the analyzers indicate the necessity of peripheral blood smear examination, which together with the CBC results provide a complete hematological picture of the patient (*9*, *10*, *13*).

A total of five analyzer flags were displayed in these two cases. Sysmex flagging interpretation guide with suggested actions helps laboratories in writing their protocols. Flags ‘RBC Abn Distribution‘ and ‘Dismorphic Population‘ are generated when the histogram pattern is abnormal. Specifically, the first one will also appear when the number of RBC < 0.5 x10^12^/L, while the second indicates multiple peaks in the RBC histogram pattern. Flags ‘Turbidity/HGB Interf?’ and ‘RBC Agglutination’ occur due to interference either in the Hb measurement channel or the RBC/Plt measurement channel. Flag ‘RBC Agglutination’ is triggered when the MCHC is greater than 400 g/L, so this is the reason why it appeared only in the first case, and ‘Turbidity/HGB Interf?’ when the MCHC is greater than 365 g/L (*14*). Analogously, the Dymind message ‘HGB Abnor./ Interfere?’ appears because of suspicious RBC or Hb, according to the scattergrams and histograms of the analyzer (*18*).

Automated hematology analyzers measure Hb directly by spectrophotometry in another channel, and since then, it is not affected by cold agglutinins (*10*, *13*). However, a decrease in Hb concentration due to massive intravascular hemolysis is typical for CIIHA. The Hb concentration of Patient 1 was still around the lower limit of the reference interval, which is probably because the patient did not develop any symptoms. Patient 2 had Hb concentration slightly below the lower limit of the reference interval. A massive drop in Hb concentration leads to serious complications like shock, circulatory arrest, organ ischemia, disseminated intravascular coagulation, acute respiratory distress syndrome, acute tubular necrosis, and death (*1*).

Acute renal failure has been observed in at least 40% of pediatric patients with CIIHA present and has been approximated with a mortality rate of 55% (*19*). In a case report published by Tao *et al.*, a 3-year-old boy with the considered diagnosis of bronchitis developed ceftriaxone-induced severe anemia, renal calculi, and cholecystolithiasis (*20*). Some adverse effects of ceftriaxone are elevated liver enzymes in 3% of patients, diarrhea in 2.7%, leukopenia in 2.1%, and hypersensitivity reactions in 1.7%. Some of the rarer (< 0.1%) but severe complications include agranulocytosis, pneumonitis, anaphylaxis, biliary lithiasis, and colitis (*21*). The most important treatment for CIIHA and DIIHA in general is discontinuation of the suspicious drug immediately. Additionally, it is recommended to avoid the causative drug, even closely related drugs, as they have been documented to cause more severe hemolytic anemia the second time (*3*).

## What YOU should / can do in your laboratory to prevent such errors

Although DIIHA is rare, drugs like ceftriaxone may be responsible for RBC agglutination even before any symptoms appear. Specific laboratory protocols for cases like these will easily detect and prevent errors caused by RBC agglutination, but it is significant to suspect. Guidelines from analyzer manufacturers help each laboratory in writing those protocols. A timely reaction can stop the development of hemolytic anemia, and hematology analyzers can assist in that by checking their comments in the blood smear. However, better knowledge of their potential and limits is crucial. Besides, it is necessary to get information on the patient’s therapy, while clinicians should be aware of possible severe complications during the administration of frequently used antibiotics in clinical practice.

## Data Availability

The data generated and analyzed in the presented study are available from the corresponding author on request.
